# Multi-phase seismic source imprint of tropical cyclones

**DOI:** 10.1038/s41467-021-22231-y

**Published:** 2021-04-06

**Authors:** Lise Retailleau, Lucia Gualtieri

**Affiliations:** 1 Université de Paris, Institut de physique du globe de Paris, CNRS, Paris, France; 2 Observatoire Volcanologique du Piton de la Fournaise, Institut de physique du globe de Paris, La Plaine des Cafres, France; 3 Department of Geophysics, Stanford University, Stanford, CA USA

**Keywords:** Solid Earth sciences, Seismology

## Abstract

The coupling between the ocean activity driven by winds and the solid Earth generates seismic signals recorded by seismometers worldwide. The 2–10 s period band, known as secondary microseism, represents the largest background seismic wavefield. While moving over the ocean, tropical cyclones generate particularly strong and localized sources of secondary microseisms that are detected remotely by seismic arrays. We assess and compare the seismic sources of P, SV, and SH waves associated with typhoon Ioke (2006) during its extra-tropical transition. To understand their generation mechanisms, we compare the observed multi-phase sources with theoretical sources computed with a numerical ocean wave model, and we assess the influence of the ocean resonance (or ocean site effect) and coastal reflection of ocean waves. We show how the location and lateral extent of the associated seismic source is period- and phase-dependent. This information is crucial for the use of body waves for ambient noise imaging and gives insights about the sea state, complementary to satellite data.

## Abstract (web summary)

The authors locate the maximum seismic energy imprint and lateral extent of the seismic sources generated by Typhoon Ioke. Based on this data set, they present a new tool to shed light on the generation mechanism of secondary microseisms body waves.

## Introduction

Ocean storms generate seismic signals through coupling between ocean waves and the solid Earth^1–3^. The secondary microseism—the strongest background seismic energy of the Earth—is generated by the nonlinear interaction between pairs of sets of ocean gravity waves with overlapping frequency content and opposite directions. The period of the resulting seismic waves—between 2 and 10 s—is half the period of the involved ocean waves. Extreme events, such as tropical cyclones, are among the most efficient storms to generate secondary microseisms^4^, whose sources are well-localized. Tropical cyclones rotate counterclockwise in the northern hemisphere while moving over the ocean. As theorized by Longuet-Higgins^5^, when they overrun their previously generated waves, ocean wave–wave interactions occur in the tail of the events themselves.

The seismic sources of ocean storms were observed first by analyzing body-wave signals at seismic arrays^6–9^. Stacking the arrivals of a seismic phase recorded at an array of stations allows for increasing its signal-to-noise ratio and linking the seismic phase to its source^10^. Specific events have been identified and studied through the lens of seismology, such as hurricane Katrina^11^, typhoon Ioke^12^, hurricane Sandy^13^, and hurricane Bill^14^. A vast majority of these studies focused on the extraction of the most energetic signals, at narrow period bands, and over a limited period of time. Retailleau and Gualtieri^15^ extended these analyses and were able to seismically track the path of typhoon Ioke (2006) throughout its entire life cycle and over the whole period band of secondary microseisms. All these studies focused on retrieving the sources of compressional P waves. Using the classical beamforming analysis technique, very few studies have extracted the signal associated with shear S waves^16–19^, as they have low amplitude, often below the noise level^20^. As a consequence, there is a big gap of knowledge on the location and generation mechanisms of secondary microseism S waves.

The cross-correlation of the ambient seismic wavefield data (often referred to as “ambient noise”) has been shown to be complementary to earthquake data for imaging the Earth’s structure through surface seismic waves^21^. More recently, the cross-correlation technique has been used successfully to extract body waves and information about the deep structure of the Earth^22–24^. This technique relies on the hypothesis that the sources of ambient noise are equipartitioned. The nonuniform distribution of sources and the lacking comprehension of the signal are the main limitations for imaging the Earth through ambient-noise body waves^25^. Indeed, errors and uncertainties associated with source location and mechanisms affect cross-correlation measurements^26^ and do not allow for discriminating between source- or structure-originating velocity variations^27^. In particular, S waves, which are the commonly used seismic phases for deep Earth imaging^28^, are challenging to retrieve, with significant uncertainties associated with them. As a consequence, the retrieval of body-wave phases for deep Earth imaging so far has focused mostly on P waves. Knowledge of the sources is crucial, both in terms of location and lateral extent, to image the subsurface reliably.

**Fig. 1:**
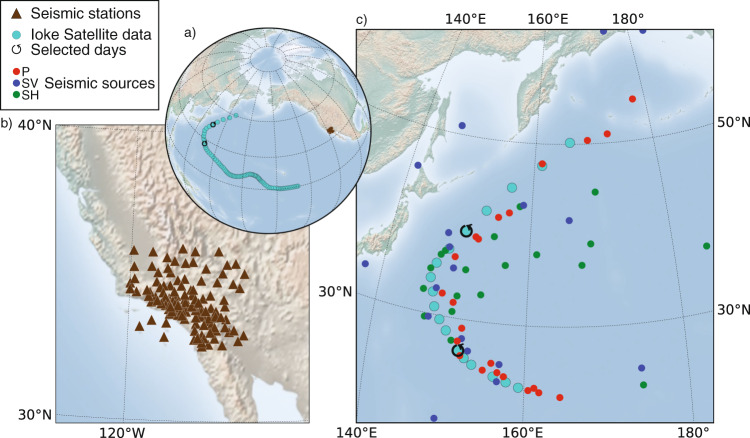
Typhoon Ioke, seismic stations, and associated body-wave sources. **a** Satellite track of typhoon Ioke (cyan) and Southern California stations (brown). **b** Zoom over the Southern California seismic stations used in this study for locating the sources of typhoon Ioke. **c** Locations of body-wave seismic sources in the 4–6 s period band (P-wave sources are in red, SV-wave sources in blue, and SH-wave sources is green). The two circled arrows in panels **a** and **c** mark the two locations of the typhoon studied in this paper.

In this study, we focus on extracting the complete body-wave (compressional and shear) imprint of typhoon Ioke (Fig. 1a). Typhoon Ioke was one of the longer-lasting tropical cyclones in the Pacific Ocean and the most intense ever recorded in the Central Pacific. It occurred in August–September 2006, and it is one of the few tropical cyclones to reach Category-5 status on the Saffir–Simpson Scale in the Central and North Pacific Ocean. We do not only focus on locating the maximum seismic energy imprint, but also on evaluating the lateral extent of the sources. We use the Southern California seismic data^29^ (Fig. 1a, b) to identify and locate the seismic sources of typhoon Ioke using the back-projection method^10^ developed by Retailleau et al.^30,31^. This method was adapted to study P-wave sources of typhoon Ioke by Retailleau and Gualtieri^15^.

## Results

### Inferring the seismic sources

While Retailleau and Gualtieri^15^ focused on retrieving the track of the event by locating P-wave sources, here we focus on the latest part of the event, during which the typhoon made the tropical–extratropical transition (around 30 ^∘^N), from September 2 to 6 2006, before becoming an extratropical storm. In particular, we extract the body-wave phases generated by the typhoon from seismograms recorded by 129 stations in Southern California^29^. We perform back-projection^10^ analysis in the rotated radial–transverse-coordinate system to extract the P, SV, and SH seismic phases and enhance the quality of the signals. Further details regarding satellite and seismic data can be found in Supplementary Note 1. Further details about the data analysis technique are in “Methods”. As verified by Retailleau et al.^30,31^, this method is able to correctly locate earthquake sources worldwide. To further assess the array response and estimate the influence of the Earth’s 3D structure and topography on our measurements, we perform classical beamforming analyses^10^ on synthetic signals obtained by employing the SPECFEM3D_GLOBE package^32,33^. We use a single source at the location of the typhoon and three different Earth models, and compare the beamforming results of P, SV, and SH waves (for more details, see Supplementary Note 2). Our analysis of synthetic data reveals that the presence of topography does not have a significant effect on the retrieval of sources of P, SV, and SH waves. The retrieval of P-wave sources is not influenced either by 3D heterogeneities (Supplementary Fig. 1). The influence of the 3D Earth’s structure on the retrieval of SV (Supplementary Fig. 2) and SH (Supplementary Fig. 3) waves is more difficult to assess as they are poorly generated in the absence of heterogeneities, coherently with recent findings on Love waves^34^. Further studies will be needed to quantify the effect of 3D heterogeneities on the generation and propagation of S waves.

Figure 1c shows the location of the maximum seismic energy associated with P, SV, and SH waves during the typhoon tropical-extratropical transition in the period band 4–6 s. As observed by Retailleau and Gualtieri^15^, P-wave sources (red dots) follow the typhoon track (cyan) during the entire life cycle of the event, confirming the generation theory proposed by Longuet-Higgins in 1953^5^. Shear-wave sources (blue and green dots) are more scattered and tend to follow the track only along portions of it or their amplitude is too small to be observed. Indeed, SV-wave sources are located close to the event before the tropical–extratropical transition, while they get scattered afterward. SH-wave sources are located close to the typhoon track only between 30^∘^ and 40^∘^N, while they are scattered at lower and higher latitudes. Among these three body-wave phases, P waves are confirmed to be the best proxy for typhoon track.

Ambient-noise sources generated by storms in the ocean are not earthquake-like point sources. In order to study the lateral extent of the source and better understanding the source mechanisms, we focus our analysis on two periods of time (circled arrows in Fig. 1a, c), before and after the tropical–extratropical transition. During these two periods of time, Ioke’s dynamic was very different and the event was located in two very different environments. On September 3 at 18:00 UTC, the typhoon was a Category 2 event on the Saffir–Simpson scale with a maximum sustained wind speed of 48.87 m/s. It was located far away from the coast in a deep-water environment. Contrarily, on September 5 at 18:00 UTC, the typhoon was weaker and classified as a tropical storm with a maximum sustained wind of 28.29 m/s. At that time, Ioke approached the Japanese coast, though still in a deep-water environment.

### Comparing observations with models

Recent developments in modeling the seismic sources of secondary microseisms from ocean-wave action models^35–37^ allowed to make predictions of the location of the seismic sources of P- and SV waves^20^ and to simulate the amplitude of P waves^38^. However, important questions are still open, such as the effect of the reflection of ocean waves at the coast and the effect of the bathymetric roughnesses on the source location and lateral extent.

In a first effort to compare both location and lateral extent of observed and simulated body-wave sources, we compute synthetic P- and SV-wave sources using the ocean-wave model WAVEWATCH III^37^. The model returns the pressure power spectral density (PSD) due to the ocean wave–wave interaction which can occur offshore, far away from continents, or close to the coast, due to the reflection of ocean waves. Coastal reflection is not well-constrained as it depends on many factors, such as the shape of the coast. The model allows for excluding coastal reflection or for including it, up to a maximum coastal reflection coefficient of 10%^37^. Like in Retailleau et al.^31^, we use this value to include sources due to coastal reflection (see Supplementary Note 3, for a comparison of the sources with and without coastal reflection). Moreover, we correct the pressure PSD for the ocean site effect^20^, which accounts for the reverberation of P waves in the water column (see “Methods” and Supplementary Note 3).

**Fig. 2:**
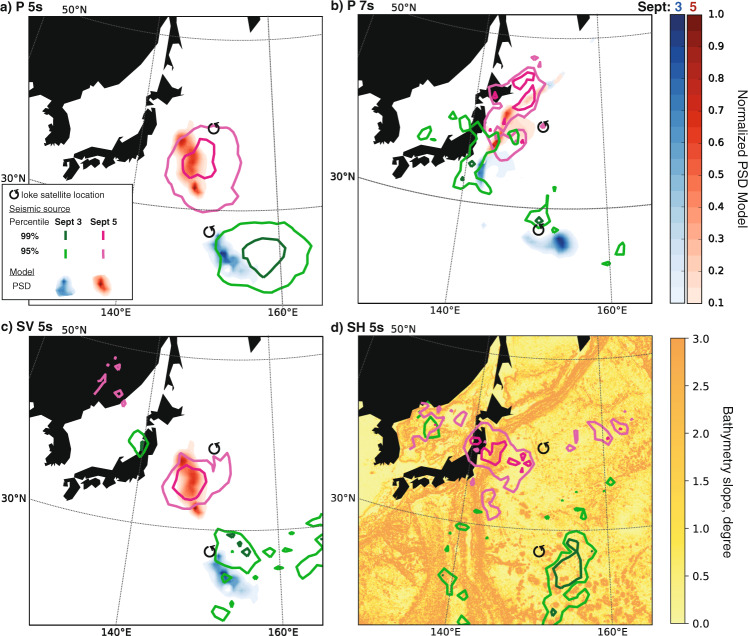
Observed and modeled extended seismic sources of typhoon Ioke. Compressional P-wave sources are shown on the top two panels—at (**a**) 5 s and (**b**) 7 s—while shear-wave sources are shown on the bottom two panels—(**c**) SV-wave sources at 5 s, and (**d**) SH-wave sources at 5 s. Observed seismic sources generated by typhoon Ioke are denoted by contour lines (green contours for sources on September 3 and pink contours for sources on September 5). Modeled P- and SV-wave sources (panels **a**–**c**) represent the pressure PSD in the presence of coastal reflection, modulated by the ocean site effect, and they are shown as colored shadows (blue for sources on September 3 and red for sources on September 5). Observed SH-wave sources in panel **d** are compared to the slope of the bathymetry.

At 5-s period (Fig. 2a), the seismic energy of P waves (contour lines) is located in the tail of the typhoon both on September 3 and 5 (green and pink contour lines, respectively). The modeled P-wave sources at 5-s period (blue and red shadows for September 3 and 5, respectively) predict well the observed sources. However, synthetic sources appear to be confined closely behind the event, especially on September 3, while the observed seismic energy covers a larger area away from the typhoon. Both observed and synthetic sources show energy along the track of the typhoon, in the open ocean, and no clear effect of the coast is observed. The observed source is very well resolved (see the results of the bootstrap analysis in Supplementary Note 4, Supplementary Fig. 5a).

At 7-s period, the observed and synthetic energy associated with P waves are not only located in the tail of the typhoon but also along the coast of Japan (Fig. 2b). Both observed and synthetic sources show a coastal component either on September 3, when the event is still in the open ocean and on September 5, when it approaches the coasts of Japan. To understand what caused the sources close to the coasts of Japan, we compute synthetic sources with and without coastal reflection and ocean site effect (Supplementary Note 3 and Supplementary Fig. 4). Because ocean waves are dispersive by nature, the long-period waves are expected to move ahead and reach the coast before the short-period waves. However, we verified that the contribution of the dispersion of ocean gravity waves to the pressure PSD associated with the nonlinear ocean wave–wave interaction is several orders of magnitude smaller than the sources in the open ocean (Supplementary Fig. 4a). Both coastal reflection and ocean site effect contribute to the emergence of these sources. While the coastal reflection is likely the dominant generation mechanism for these sources (Supplementary Fig. 4c), the ocean site effect (Supplementary Fig. 4b) contributes to reshaping the lateral extent of the source (Supplementary Fig. 4d). It is the ocean site effect that allows the synthetic sources to assume a northwest–southeast shape, similarly to the observed sources. We note, however, that the observed seismic sources are, on average, closer to the Japanese coast than synthetic sources, potentially due to the uncertainties in constraining the ocean-wave reflection coefficient in our modeling.

### Shear-wave analysis

Shear-wave sources remain largely unexplained so far, because of the low amplitude and signal-to-noise ratio of these seismic phases. We extract shear-wave energy at 5 s for the two selected periods of time (Fig. 2c, d). Observed and synthetic sources associated with SV waves show a good match on September 5 (Fig. 2c). However, the orientation of the lateral extent of the synthetic source is perpendicular to the orientation of the observed source, which follows the typhoon track. On September 3, observed and synthetic sources show a slightly different location. This is likely because the observed source is less resolved (Supplementary Fig. 5b). A second source is observed further offshore, but it is likely not associated with the typhoon (see Supplementary Note 4).

Observations of SH-wave sources are extremely rare^16–19^. We extract the signature of SH waves at 5-s period and back-project their sources (Fig. 2d). The location of the sources is robust (see Supplementary Note 4 and Supplementary Fig. 5c). On both days, the source is located in the tail of the typhoon. In the absence of 3D heterogeneities and seafloor topography, pressure sources cannot generate any SH waves. The mechanism for the generation of SH waves is currently unknown. One hypothesis is that bathymetric inclines allow for splitting the force pressure into vertical and horizontal components. The horizontal component would be responsible for the generation of SH waves. In order to test this hypothesis, we compare the observed sources to the bathymetry slope. Overall, we observe that the slope of the bathymetry is quite gentle and does not exceed a few degrees. On September 3, the source is located on a relatively flat bathymetric area, with the only presence of seamounts. On September 5, the source is located close to the coast and above the Japan trench, where the bathymetry slope gets steeper than the previous case. At 5-s period, we do not observe P- and SV-wave sources close to the coast (Fig. 2a, c), but only SH-wave sources. This evidence is confirmed by synthetic simulations (Supplementary Note 2 and Supplementary Fig. 3), which revealed that SH waves from a point source at the location of the event only emerge in the presence of 3D heterogeneities, regardless of the bathymetry at the source region. This suggests that a possible explanation for SH waves is scattering and focusing-defocusing at heterogeneities within the Earth, similarly to what observed for Love waves^34^. We do not observe any correlation between the location of the source and the thickness of the underneath sediments, suggesting the generation of SH waves may occur deeper into the Earth (see Supplementary Note 5 and Supplementary Fig. 7).

## Discussion

To go deeper into the generation mechanisms of the three seismic phases in terms of location and lateral extent of the sources, we compare the sources of P, SV, and SH waves as observed on September 3, when Ioke was a Category 2 tropical cyclone.

**Fig. 3:**
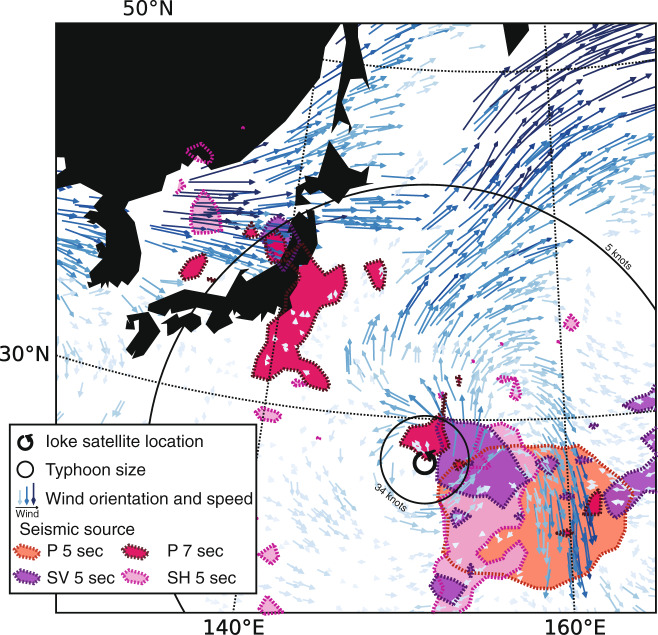
Comparison of location and lateral extent of body-wave sources. Map of P-, SV-, and SH-wave source imprints on September 3 at 18:00 UTC. For reference, we show the wind field direction and strength (arrows), and the size of the typhoon defined as the radius that incorporates wind speeds larger than two given thresholds (5 and 34 knots).

Observations of the three body-wave phases on September 3 allow us to make comparisons of the location and lateral extent of the sources (Fig. 3). At 5-s period, sources of P, SV, and SH waves are very close to each other and partially overlap. They are located in the same area in the tail of the typhoon. The source of P waves shows the largest lateral extent, possibly due to the ocean site effect at the source, which is stronger for P than SV waves (“Methods” and Fig. 5). It could also be due to the fact that signals associated with P waves are more energetic and less attenuated at the receivers, yielding to a more efficient back-projection of the source. The region area where P, SV, and SH waves at 5-s period are generated lies between 34 and 5-knots wind-threshold size (see Supplementary Note 1, for more information about typhoon size and wind field). As observed in Fig. 2, the source of P waves at 7 s is the only one on September 3 whose energy is mostly concentrated along the coast, highlighting the predominance of a different mechanism. This source is far away from the typhoon and close to the 5-knots wind-threshold size. We observe that the sources of P, SV, and SH waves at 5-s period are located in the South-East quadrant where the wind speed above the ocean (arrows in Fig. 3) is high. On the other hand, the P-wave source at 7-s period is located in a region of low wind speed, further enhancing the predominance of generation mechanisms other than the direct coupling between wind field and surface ocean waves, e.g., ocean-wave coastal reflection.

Sources of secondary microseism shear body waves have been poorly studied so far, and only the location of the maximum energy has been found in a few cases^15–19^. Retailleau and Gualtieri^15^ analyzed the P waves generated by typhoon Ioke in the secondary microseism band. They showed that the seismic sources of P waves follow Ioke’s track as soon as the typhoon gets strong enough and until it dies out as an extratropical storm. Following the theory developed by Haubrich^39^, they also showed that there is a cut-off period for the generation of seismic sources that is related to the propagation speed of the typoon. The comparison of the lateral extent and source location of P, SV, and SH waves represents a new observation that sheds light on the generation mechanism of secondary microseisms body waves. The location of the sources and the generation mechanism of secondary microseisms vary significantly with frequency. In the specific case of Typhoon Ioke, we observe the predominance of coastal reflection mechanisms at the long period (*T* = 7 s) and ocean wave–wave interaction in a deep-water environment at the short period (*T* = 5 s). We also observe that dispersion of ocean gravity waves alone cannot explain sources close to the coast, both in terms of location and shape. This is evidence that the source area does not necessarily coincide with the generation area, but it is reshaped by the ocean site effect (Supplementary Note 3 and Supplementary Fig. 4). We do not observe any major bathymetric features, or a particularly thick sedimentary layer, in the source area of SH waves, indicating that they may generate at lateral 3D heterogeneities deep into the Earth.

Those observations are crucial for imaging the Earth’s structure with secondary microseisms. For example, the distance between the centroid of the P-wave sources at 5 s and 7 s (Figs. 2 and 3) is about 15°. Assuming the same location for both of them yields to an error in the travel time and velocity-variation measurements of 30 s and 7.6% for P waves, and 60 s and 6.3% for S waves. If not properly taken into account, these large time shifts may be mistaken for structural variations, as pointed out by Kedar^36^.

It is well known that caution is needed to use secondary microseisms as a proxy for assessing the sea state, as the seismic sources of ocean storms do not correspond, in most cases, to the location of the maximum wind field or wave height. On the other hand, the seismic sources of storms are the proxy for identifying the portions of the ocean surface where the nonlinear ocean wave–wave interaction occurs efficiently. For example, Retailleau and Gualtieri^15^ showed that the P-wave sources associated with typhoon Ioke could not be extracted on the first portion of the track, but they could be identified during the extratropical late portion of the track. In both cases, the event was weak, in the process of forming or disappearing, but only in the latter case, the ocean wave–wave interaction occurred efficiently to generate seismic sources. Seismic observations can thus be informative about the sea-state conditions for which the wave interactions occur and the dependence of the sea state on the wind field blowing over the ocean^40,41^. Seismic sources of ocean storms have the potential to shed light on the coupling between the ocean and the atmosphere, in addition to the solid Earth.

## Methods

### Seismic data analysis

We use the time series recorded between September 2 and 6, 2006 by a network of 129 stations in Southern California (Fig. 1a, b), made available by the Southern California Earthquake Center^29^. The Southern California network is wide enough to extract P, SV, and SH phases at the selected periods and homogeneous enough to resolve well the source location for the two selected periods of time. We exclude stations in Central California to keep a more homogeneous station distribution.

**Fig. 4:**
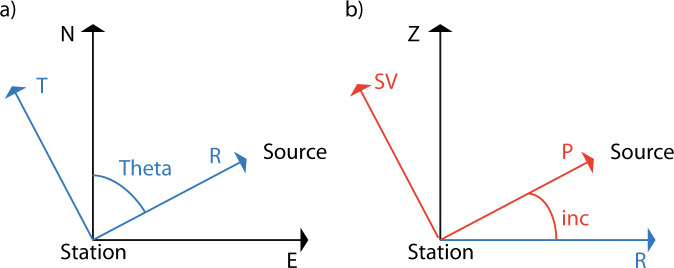
Rotation to the (P, SV, T) coordinate system. Rotation process to convert the seismograms from the (N, E, Z) coordinate system first to **a** the (R, T, Z) coordinate system where "Theta" denotes the azimuth and then to **b** the (P, SV, T) coordinate system where "inc" is the incident angle of the P wave.

The data were processed using the python toolboxes *numpy*, *scipy*, and *Obspy*^42^. The seismograms are deconvolved with the instrument responses to get ground velocity seismograms. The signals are also downsampled to 2 Hz. The seismograms are then rotated from the (N, E, Z) coordinate system to the (P, SV, T)—where T stands for the transverse-coordinate system (Fig. 4). Finally, the seismograms are filtered using a Butterworth band-pass filter in the period bands 4–6 s (Fig. 1c), 4.9–5.1 s (Figs. 2 and 3), and 6.9–7.1 s (Figs. 2 and 3).

The back-projection of the seismic source follows the process developed by Retailleau et al.^30^ and Retailleau et al.^31^ and applied on typhoon Ioke  by Retailleau and Gualtieri^15^. For each 3-h time window analyzed, we perform a grid search of the potential sources around the typhoon’s satellite location (20^∘^ × 20^∘^ in longitude and latitude, and a grid spacing of 0. 5^∘^). For each of these locations, we compute a vespagram^10^ from the data window and extract the energy that corresponds to the expected arrival slowness predicted in the 1-D Earth model IASP91^43^ for the P, SV, and SH waves on the P, SV, T components, respectively^42,44^. Selecting energy precisely along the expected velocity permits to enhance the useful signal and extract high signal-to-noise ratio information. The final result is a normalized back-projection of the source.

### Modeling the sources of P and Sv waves

Secondary microseism sources are generated by the interaction of ocean gravity waves at the surface of the ocean^45^. We model the power spectral density (PSD) of the pressure field generated by ocean wave–wave interaction by using the numerical ocean-wave model WAVEWATCH III^37,46^. The PSD of the pressure field (Pa^2^/Hz) is defined as

1 $${F}_{p}(f,\theta ,\phi )={(2\pi )}^{2}\,\frac{{\rho }_{w}^{2}\,{g}^{2}f\,{E}^{2}({f}_{w})I({f}_{w})}{{\mathrm{d}}S(\theta ,\phi )}$$

where *f* is the seismic frequency, *θ* is the colatitude, *ϕ* is the longitude, *ρ*_*w*_ is the density of the water (assumed constant), *g* is the gravity acceleration. The elementary surface is ${\mathrm{d}}S={R}^{2}\sin \theta \,{\mathrm{d}}\theta \,d\phi$, where *R* is the radius of the Earth. The factor *E*(*f*_*w*_) is the PSD of the sea surface elevation (m^2^/Hz), and *I*(*f*_*w*_) is the non-dimensional ocean gravity wave energy distribution as a function of frequency, integrated over the ocean-wave azimuth.

The ocean acts as a waveguide for P waves, which are multiply reflected between the surface of the ocean and the seafloor. At each reflection point at the seafloor, P and SV waves are generated by energy conversion and transmission. The effect of the multiple reflected P waves in the ocean on the wavefield beneath the seafloor is called ocean site effect^20^. Longuet-Higgins^45^ worked out the ocean site effect on Rayleigh waves traveling beneath the seafloor. He observed that the ocean site effect is depth- and frequency-dependent. Notably, at *T* = 5-s period, the fundamental mode of Rayleigh waves is mostly amplified at ~2–3 km water depths, while the first overtone experiences the largest resonance in much deeper oceanic environments, at about 5–6 km (see ref. ^46^, their Fig. 2). For body waves, Gualtieri et al.^20^ found that there are several peaks of amplification, corresponding to different depths and frequencies. At *T* = 5 s, the acoustic resonance on P and SV waves happens at similar depths, about 2–3 km and 5–6 km (see ref. ^20^, their Fig. 3).

**Fig. 5:**
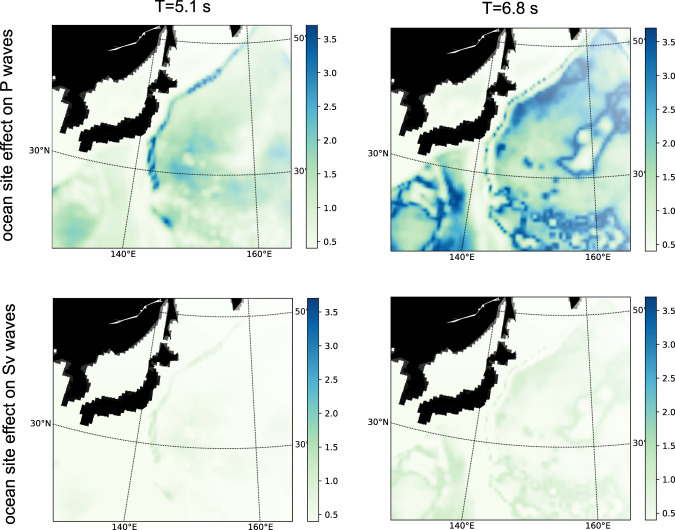
Ocean site effect on P and Sv waves. Ocean site effect on P (top) and Sv (bottom) waves at 5.1-s (left) and 6.8-s (right) period.

Sources of P and SV waves can be computed by multiplying the PSD of the pressure (Eq. (1)) with the ocean site effect on P and SV waves, respectively. The ocean site effect on body waves varies with frequency, ocean depth, and epicentral distance (ref. ^20^, their Eqs (4) and (12)). We compute the ocean site effect on P and Sv waves at the same frequencies of the ocean-wave model and considering the epicentral distance between the typhoon location and the average location of the stations (Fig. 1b). Figure 5 shows the ocean site effect on P and S waves at *T* = 5.1 s and *T* = 6.8 *s*. As already observed by Gualtieri et al.^20^, the ocean site effect on Sv waves has a similar spatial pattern of the ocean site effect on P waves, but it is characterized by a significantly lower amplitude.

## Supplementary information

Supplementary Information

## Data Availability

The seismic dataset used for this study can be accessed at the Southern California Data Center^29^ through the *Obspy* toolbox. Center locations of Typhoon Ioke is taken from the Joint Typhoon Warning Center (JTWC) best track dataset^47^ (https://www.metoc.navy.mil/jtwc/jtwc.html?best-tracks). We used the wind field from the Cooperative Institute for Meteorological Satellite Studies (CIMSS) (http://tropic.ssec.wisc.edu/tropic.canned.php). The output of the ocean-wave model can be found at ftp://ftp.ifremer.fr/ifremer/ww3/HINDCAST/SISMO/. Websites were last accessed on February 16, 2021.

## Code availability

The codes used to process the seismic data and perform back-projection of the sources are available upon request. To perform synthetic simulations of seismic wave propagation, we used the package SPECFEM3D_GLOBE, which is a freely available code through the Computational Infrastructure for Geodynamics (CIG, https://geodynamics.org/cig/software/specfem3d_globe/, last accessed February 16, 2021).
