# Supplementary material for: Multi-phase seismic source imprint of tropical cyclones
Source: Nat Commun. 2021 Apr 6;12:2064. doi: 10.1038/s41467-021-22231-y (PMC8024386; doi:10.1038/s41467-021-22231-y)
Supplement: Supplementary file 1 — Supplementary Information [file 41467_2021_22231_MOESM1_ESM.pdf]

Supplementary Information for:
Multi-phase seismic source imprint of tropical cyclones

Lise Retailleau[1,2*] and Lucia Gualtieri,[3*]

[1] Université de Paris, Institut de physique du globe de Paris, CNRS, F-75005 Paris, France

[2] Observatoire Volcanologique du Piton de la Fournaise, Institut de physique du globe de Paris, F-97418 La Plaine des Cafres, France

[3] Stanford University, Department of Geophysics, Stanford, CA 94305-2215, USA.

*E-mail: retailleau@ipgp.fr, gualtieri@stanford.edu.

1 Supplementary Note 1

Ioke track and size, wind field and bathymetry data

We use track and size of Typhoon Ioke as recorded every 6 hours from satellites. Center locations of Typhoon Ioke is taken from the Joint Typhoon Warning Center (JTWC) best track data set (*5*) (`http://www.usno.navy.mil/NOOC/nmfc-ph/RSS/jtwc/best_tracks/`). The size is defined as the radius that incorporates wind speeds larger than a given threshold. Two tropical-cyclone data sets are used to identify Ioke size at two different thresholds: the JTWC best track data set with a threshold at 34-kt (1 kt = 0.514 m/s) winds and a data set built using storm-centered infrared imagery (*8, 9*) with a threshold at 5-kt winds.

The wind field used in Figure 3 is taken from the Cooperative Institute for Meteorological Satellite Studies (CIMSS) (`http://tropic.ssec.wisc.edu/tropic.canned.php`), as measured by Multifunctional Transport Satellites (MTSAT). Notably, we use the MTSAT measurements of the cloud-drift wind field over the ocean surface, where the ocean-atmosphere coupling occurs, and ocean surface gravity waves are generated.

We use ETOPO1 (*1*) (Figure 3 in the main text) to compare SH-wave sources with the bathymetry. ETOPO1 has a resolution of 1 arc-minute, which is about 1.82 km at the equator and 1.13 km at the poles. This high resolution allows us to identify all major bathymetric roughnesses features that may contribute to the generation of SH waves.

2 Supplementary Note 2

The effect of topography and 3D Earth structure on the beamforming analysis

We perform beamforming analysis (*14*) on synthetic seismograms to observe the effects of topography and Earth's structure on the body-wave seismic phases. We use SPECFEM3D_GLOBE (*10, 11*) and perform three-component numerical simulations on a $90° \times 90°$ chunk of the Earth. Simulations are accurate down to 6.8 s, with 640 spectral elements along each side of the chunk. We employ a single vertical force at the location of the typhoon and we record the seismic signals at the 129 stations of the Southern California Seismic Network used in this study. The source is a vertical unitary force at the Earth's surface. Topography and 3D heterogeneities are switched on and off to test their effects. For our 1D model, we use a smoothed version of the ETOPO2 bathymetry and topography model (*4*), with a resolution of 4×4 min^2 (about 7.4×7.4 km), with the 1D Earth model PREM (*6*). As a 3D Earth model we use S40RTS (*13*), including the 3D crustal model Crust2.0 (*3*). We use one 3D model with bathymetry and one without.

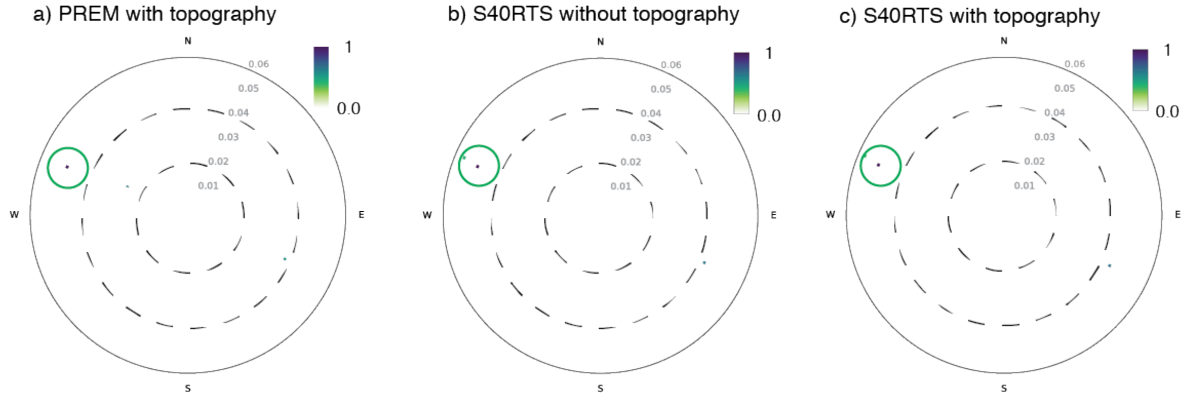

Supplementary Figure 1: Beamforming analysis of the P phase (highlighted by green circles) extracted from synthetic data computed employing three different Earth's configurations. To compare the results across Earth's models, the same normalization has been used in all cases.

We rotate the signals to the (P, SV, T) coordinate system, as we did for the data, and perform beamforming analysis in the $6.8 - 7.2$ s period band using the *array analysis* toolbox of the *Obspy* project (*12*). Supplementary Figures 1, 2 and 3 show the beamforming results on the synthetic data for the different phases and Earth's configurations. The P phase (Supplementary Figure 1) is consistent across the different Earth's configurations, and it is not influenced significantly by either bathymetry/topography or 3D structure. The presence of bathymetry/topography does not have any significant effect on SV and SH phases (Supplementary Figures 2b-c and 3b-c), which are retrieved at a nearly similar azimuth and slowness,

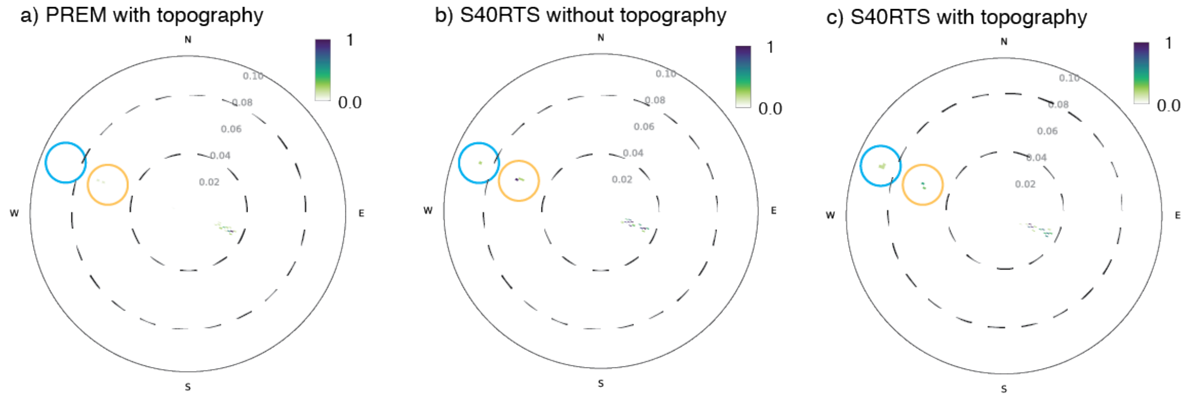

Supplementary Figure 2: Beamforming analysis of the SV phase (highlighted by blue circles) extracted from synthetic data computed employing three Earth's configurations. Orange circles highlight the SKS phase. To compare the results across Earth's models, the same normalization has been used in all cases.

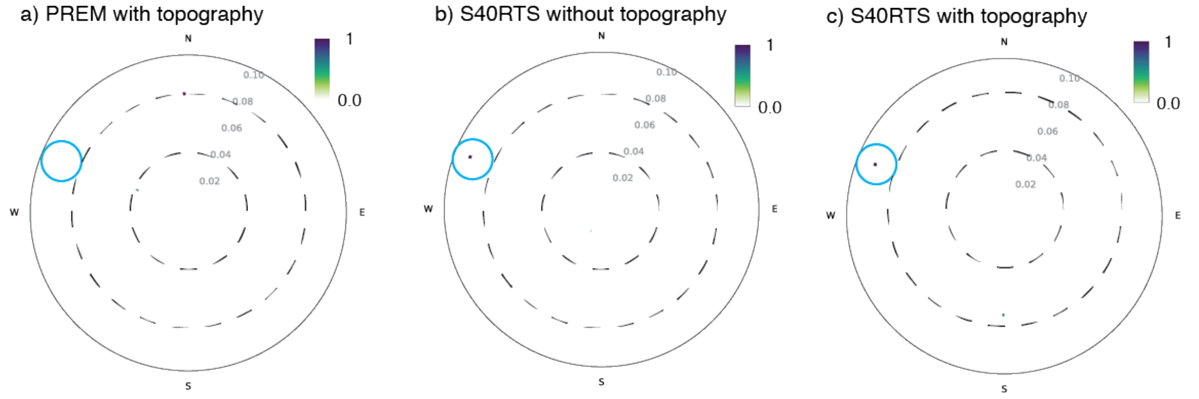

Supplementary Figure 3: Beamforming analysis of the SH phase (highlighted by blue circles) extracted from synthetic data computed employing three Earth's configurations. To compare the results across Earth's models, the same normalization has been used in all cases.

either in the presence or in the absence of bathymetry/topography. Interestingly, the absence of 3D heterogeneities prevents to observe S waves (Supplementary Figures 2a and 3a). Similarly to what was observed regarding the generation of Love waves (*7*), this may be an indication of the generation of SH waves from scattering at 3D lateral heterogeneities. Future studies will be needed to assess the influence of 3D heterogeneities on the generation and propagation of secondary microseism S waves.

3 Supplementary Note 3

Ocean site effect *vs* coastal reflection

Supplementary Figure 4 shows the effect of the coastal reflection and the multiply reflected P waves in the ocean (called "ocean site effect") on the modeled P-wave source at 7 s period. Sources are defined in terms of power spectral density of the pressure at the surface of the ocean, as computed using the ocean wave model WAVEWATCH III (*2*) (for more information, see Methods, section M2).

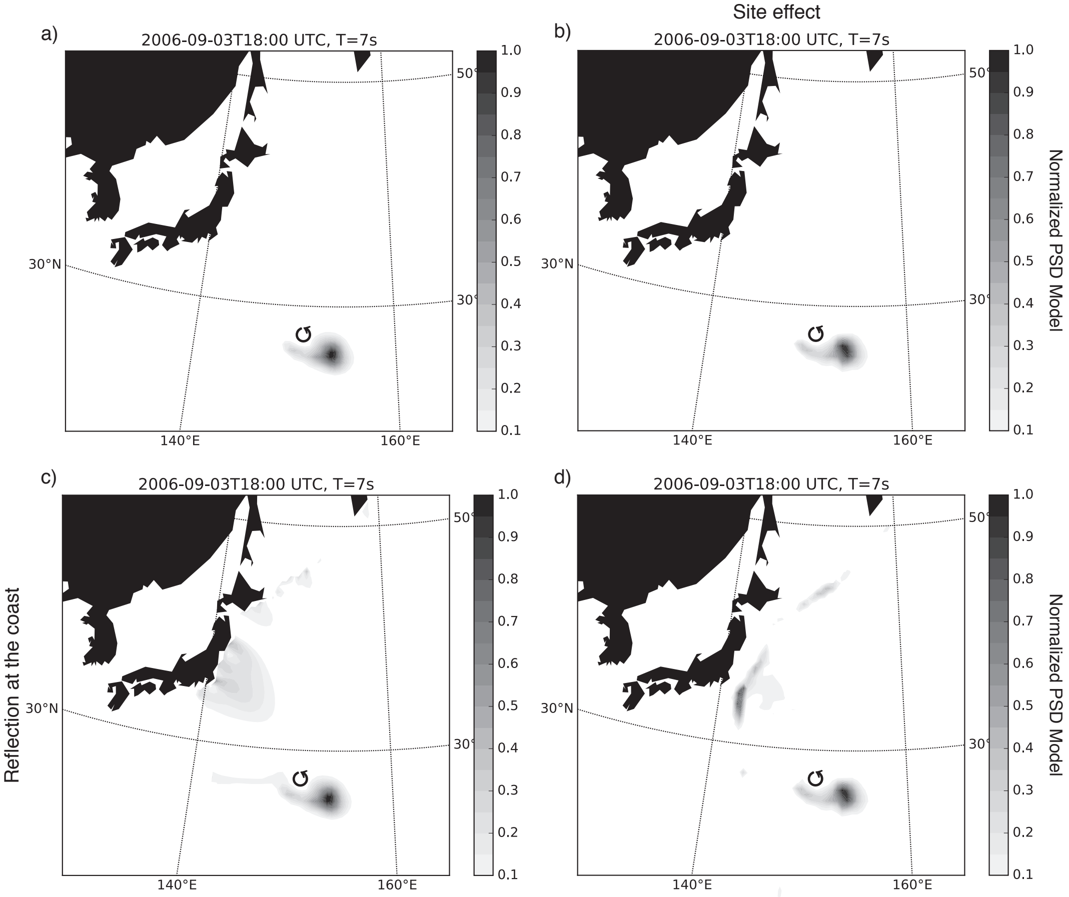

Supplementary Figure 4: Effect of the coastal reflection and the ocean site effect on the modeled sources. The panels show the pressure PSD with a) no reflection at the coast and no site effect, b) P-wave ocean site effect only, c) reflection at the coast only, d) reflection at the coast and P-wave ocean site effect.

When none of those effects are applied, a source defined in terms of pressure PSD is located

close to the typhoon (Supplementary Figure 4a). Adding the ocean site effect alone does not modify the source shape and location (Supplementary Figure 4b). Adding 10% of coastal reflection yields some energy close to the coast (Supplementary Figure 4c), but its shape does not correspond to the observed seismic source (Figure 2b). Finally, the combination of those two effects leads both to energy close to the typhoon, and energy along the coast (Supplementary Figure 4d). The modeled source in this last case is similar to the observed one (Figure 2b). While the addition of the reflection at the coast appears to have the largest effect, the ocean site effect defines the shape of the energy close to the coast, making it similar to the seismic source extracted from the data (Figure 2b).

4 Supplementary Note 4

Bootstrap and robustness of the source location

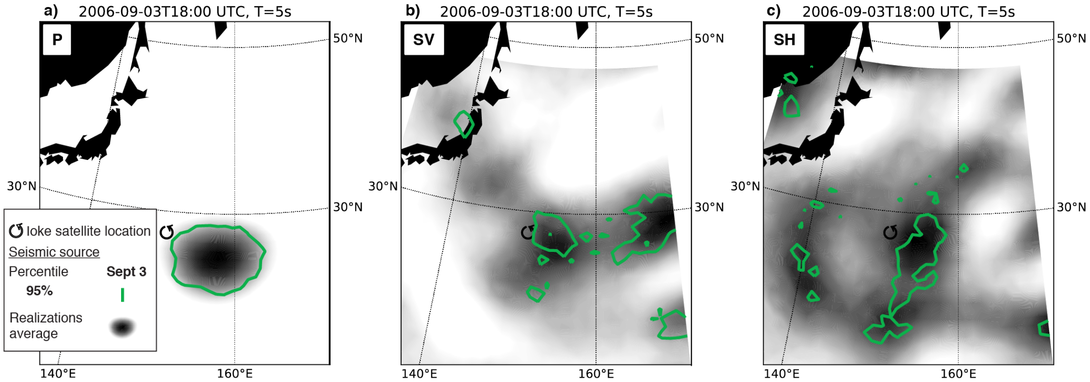

Supplementary Figure 5: Normalized average of the bootstrap realizations. The green contours represent observations, shown as the 95th percentile of the seismic sources in Figure 2c.

We perform 99 bootstrap realizations of the seismic source location to determine the robustness of our analysis. In each realization, we use 96 random stations (75% of the total number of stations) to locate the seismic source. Supplementary Figure 5 shows the average of the 99 realizations for the three phases (gray scale) superposed to observations (green contours) in Figure 2c. The P phase seismic source is very well recovered (Supplementary Figure 5a). The SV and SH phases are noisier, but the maxima of the bootstrap are located where the sources are observed (Figure 2). In particular, the SH phase (Supplementary Figure 5c) shows a dominant source consistent with observations (Figure 2), although secondary, low-energy, sources are observed through bootstrap analysis. The SV-phase bootstrap result exhibits, in addition to the main source consistent with observations, a second robust strong source away from the typhoon (Supplementary Figure 5b). As Supplementary Figure 6 shows, the ocean wave model does not

exhibit any energy at that location. On the other hand, the wind field above the ocean (arrows in Supplementary Figure 6) indicates some activity north of the area, which may suggest the presence of an extra-tropical storm, not captured by the ocean wave model, able to generate ocean wave-wave interaction.

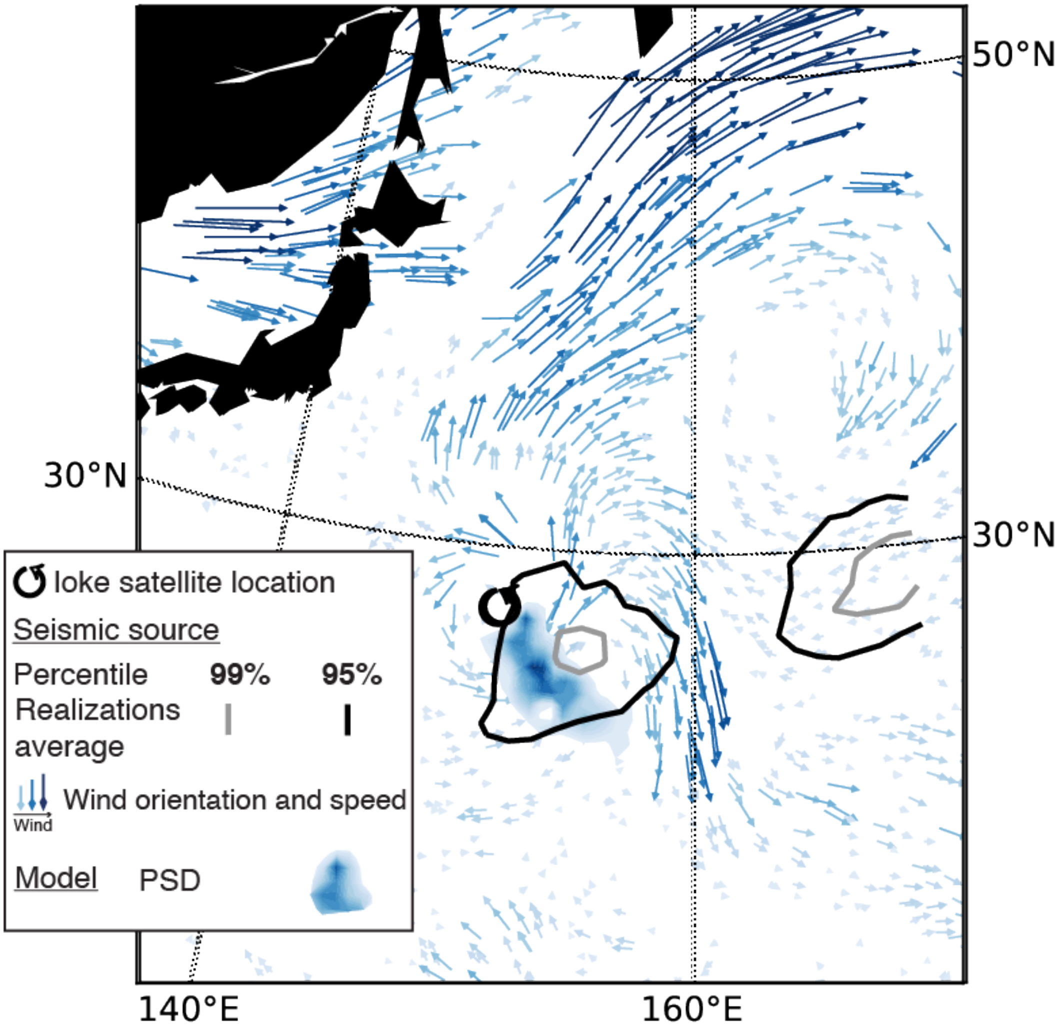

Supplementary Figure 6: The black and grey curves represent the 95th and 99th percentile of the average of the realizations of the bootstrap on the SV phase. The blue contours represents the model with site effect and reflection at the coast as in Figure 2. The blue arrows represent the wind orientation and speed as in Figure 3.

5 Supplementary Note 5

Potential influence of the sedimentary layer on the SH-phase generation

In Supplementary Figure 7, we compare the sources of SH waves obtained for September 3 at 5-s period to the thickness of the sedimentary layer in the region (*3*). A co-location between SH sources and thick sediments could be a proxy for ascribing the generation of SH waves to reverberations inside the sedimentary layer. We observe no co-location between the dominant SH source and areas with thick sediments, indicating that the sedimentary layer might not be the dominant, or the unique, contributor for SH waves.

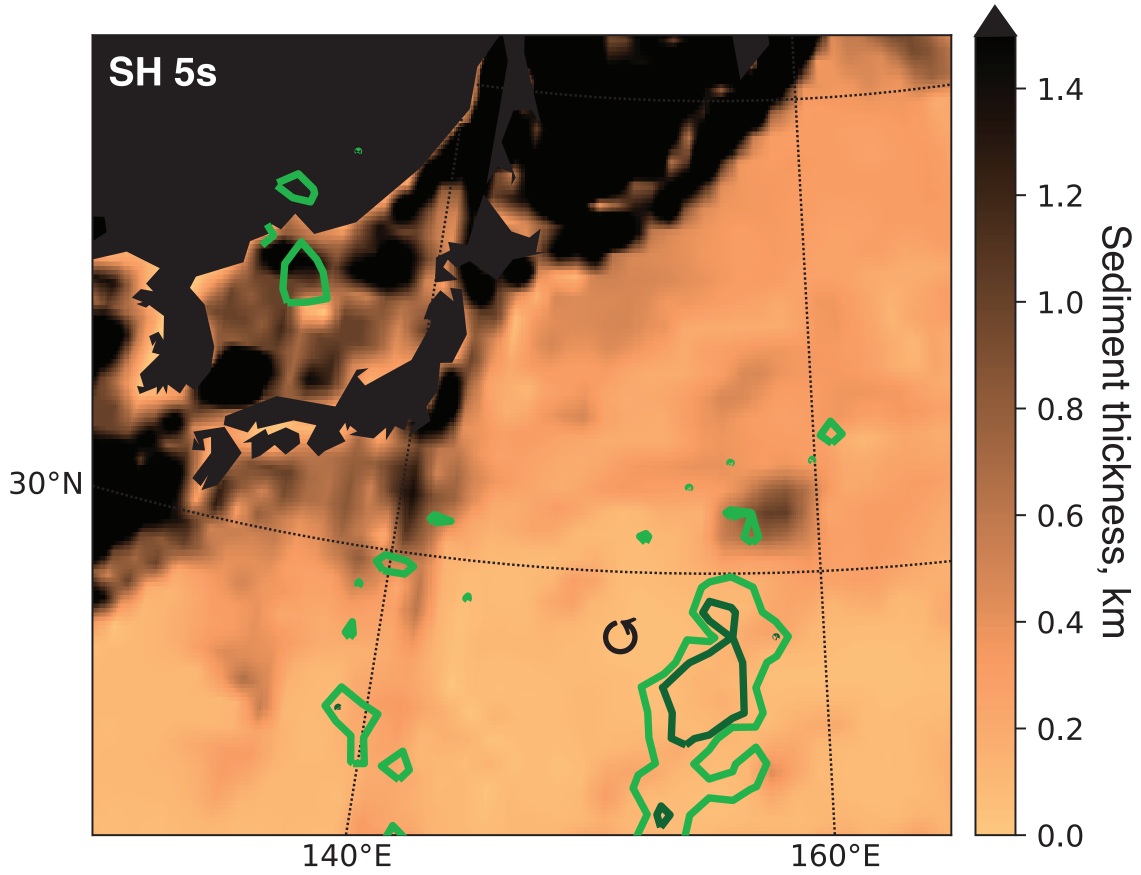

Supplementary Figure 7: Sources of SH waves at 5 s on September 3 from Figure 2d (contour lines) compared to the thickness of the sedimentary layer (color scale). Dark and light green contour lines indicate the 95th and 99th percentiles, respectively.

Supplementary References

1. C. Amante and B. W. Eakins. ETOPO1 1 Arc-Minute Global Relief Model: Procedures, Data Sources and Analysis. *NOAA Technical Memorandum NESDIS NGDC-24*, pages 1–19, 2009.

2. Fabrice Ardhuin, Eleonore Stutzmann, Martin Schimmel, and Anne Mangeney. Ocean wave sources of seismic noise. *Journal of Geophysical Research: Oceans*, 116(C9), 2011.

3. Chantal Bassin. The current limits of resolution for surface wave tomography in North America. *EOS Trans. AGU. 81: Fall Meet. Suppl., Abstract*, 2000.

4. National Geophysical Data Center. 2-minute gridded global relief data (ETOPO2v2), 2006.

5. Jan-Hwa Chu, Charles R Sampson, Andrew S Levine, and Edward Fukada. The joint typhoon warning center tropical cyclone best-tracks, 1945–2000. *Ref. NRL/MR/7540-02*, 16, 2002.

6. Adam M Dziewonski and Don L Anderson. Preliminary reference Earth model. *Physics of the earth and planetary interiors*, 25(4):297–356, 1981.

7. Lucia Gualtieri, Etienne Bachmann, Frederik J Simons, and Jeroen Tromp. The origin of secondary microseism Love waves. *Proceedings of the National Academy of Sciences*, 2020.

8. John A Knaff, Scott P Longmore, and Debra A Molenar. An objective satellite-based tropical cyclone size climatology. *Journal of Climate*, 27(1):455–476, 2014.

9. John A Knaff, Scott P Longmore, and Debra A Molenar. CORRIGENDUM: An Objective Satellite-Based Tropical Cyclone Size Climatology. *Journal of Climate*, 28(21):8648–8651, 2015.

10. Dimitri Komatitsch and Jeroen Tromp. Spectral-element simulations of global seismic wave propagation—I. Validation. *Geophysical Journal International*, 149(2):390–412, 2002.

11. Dimitri Komatitsch and Jeroen Tromp. Spectral-element simulations of global seismic wave propagation—II. Three-dimensional models, oceans, rotation and self-gravitation. *Geophysical Journal International*, 150(1):303–318, 2002.

12. Lion Krischer, Tobias Megies, Robert Barsch, Moritz Beyreuther, Thomas Lecocq, Corentin Caudron, and Joachim Wassermann. ObsPy: A bridge for seismology into the scientific Python ecosystem. *Computational Science & Discovery*, 8(1):014003, 2015.

13. J Ritsema, a A Deuss, HJ Van Heijst, and JH Woodhouse. S40RTS: a degree-40 shear-velocity model for the mantle from new Rayleigh wave dispersion, teleseismic traveltime and normal-mode splitting function measurements. *Geophysical Journal International*, 184(3):1223–1236, 2011.

14. S. Rost and C. Thomas. Array seismology: Methods and applications. *Reviews of Geophysics*, 40(3):1008, 2002.
